# Regulation of Glutarate Catabolism by GntR Family Regulator CsiR and LysR Family Regulator GcdR in Pseudomonas putida KT2440

**DOI:** 10.1128/mBio.01570-19

**Published:** 2019-07-30

**Authors:** Manman Zhang, Zhaoqi Kang, Xiaoting Guo, Shiting Guo, Dan Xiao, Yidong Liu, Cuiqing Ma, Chao Gao, Ping Xu

**Affiliations:** a State Key Laboratory of Microbial Technology, Shandong University, Qingdao, People’s Republic of China; b Tianjin Key Laboratory of Radiation Medicine and Molecular Nuclear Medicine, Department of Radiobiology, Institute of Radiation Medicine of Chinese Academy of Medical Science & Peking Union Medical College, Tianjin, People’s Republic of China; c State Key Laboratory of Microbial Metabolism, Joint International Research Laboratory of Metabolic & Developmental Sciences, and School of Life Sciences & Biotechnology, Shanghai Jiao Tong University, Shanghai, People’s Republic of China; Fred Hutchinson Cancer Research Center; Department of Biology, Washington University in St. Louis, St. Louis, Missouri, USA; Institute of Pharmacy and Molecular Biotechnology, University of Heidelberg, Im Neuenheimer Feld 364, 69120 Heidelberg, Germany

**Keywords:** l-2-hydroxyglutarate, catabolism, glutarate, regulatory mechanism

## Abstract

Glutarate, a metabolic intermediate in the catabolism of several amino acids and aromatic compounds, can be catabolized through both the glutarate hydroxylation pathway and the glutaryl-coenzyme A (glutaryl-CoA) dehydrogenation pathway in Pseudomonas putida KT2440. The elucidation of the regulatory mechanism could greatly aid in the design of biotechnological alternatives for glutarate production. In this study, it was found that a GntR family protein, CsiR, and a LysR family protein, GcdR, regulate the catabolism of glutarate by repressing the transcription of *csiD* and *lhgO*, two key genes in the glutarate hydroxylation pathway, and by activating the transcription of *gcdH* and *gcoT*, two key genes in the glutaryl-CoA dehydrogenation pathway, respectively. Our data suggest that CsiR and GcdR are independent and that there is no cross-regulation between the two pathways. l-2-Hydroxyglutarate (l-2-HG), a metabolic intermediate in the glutarate catabolism with various physiological functions, has never been elucidated in terms of its metabolic regulation. Here, we reveal that two molecules, glutarate and l-2-HG, act as effectors of CsiR and that P. putida KT2440 uses CsiR to sense glutarate and l-2-HG and to utilize them effectively. This report broadens our understanding of the bacterial regulatory mechanisms of glutarate and l-2-HG catabolism and may help to identify regulators of l-2-HG catabolism in other species.

## Abstract (precis)

Glutarate is an attractive dicarboxylate with various applications. Clarification of the regulatory mechanism of glutarate catabolism could help to block the glutarate catabolic pathways, thereby improving glutarate production through biotechnological routes. Glutarate is a toxic metabolite in humans, and its accumulation leads to a hereditary metabolic disorder, glutaric aciduria type I. The elucidation of the functions of CsiR and GcdR as regulators that respond to glutarate could help in the design of glutarate biosensors for the rapid detection of glutarate in patients with glutaric aciduria type I. In addition, CsiR was identified as a regulator that also regulates l-2-HG metabolism. The identification of CsiR as a regulator that responds to l-2-HG could help in the discovery and investigation of other regulatory proteins involved in l-2-HG catabolism.

## INTRODUCTION

Glutarate is an important C5 platform chemical with many applications (1, 2). Traditionally, glutarate is produced through chemical processes that rely on petrochemical precursors (3, 4). However, environmental concerns and the depletion of oil reserves have limited the sustainable production of glutarate via chemical methods (5–7). Thus, a biobased route for glutarate production is now highly desired and the glutarate metabolic pathways are gaining worldwide attention (8–10).

Glutarate is a metabolic intermediate in the catabolism of several amino acids (such as l-lysine, l-hydroxylysine, and l-tryptophan) and aromatic compounds (such as nicotinate and benzoate) (11–14). The classic glutarate catabolism pathway is the glutaryl-coenzyme A (glutaryl-CoA) dehydrogenation pathway, where glutaryl-CoA dehydrogenase (GcdH) is the key enzyme (11). In this pathway, glutarate is first converted to glutaryl-CoA, followed by the dehydrogenation and decarboxylation of glutaryl-CoA by GcdH to produce crotonyl-CoA (15–17). Crotonyl-CoA can be converted to two molecules of acetyl-CoA, which are then channeled into the tricarboxylic acid (TCA) cycle (11, 18). Recently, a glutarate hydroxylation pathway with glutarate hydroxylase (CsiD [carbon starvation-induced protein]) and l-2-hydroxyglutarate (l-2-HG) oxidase (LhgO) as its key enzymes was identified in both Pseudomonas putida (19) and Escherichia coli (20). CsiD is capable of converting glutarate and 2-ketoglutarate (2-KG) into l-2-HG and succinate. The l-2-HG produced is subsequently converted to 2-KG by LhgO. These two pathways have been found to cooperate in glutarate catabolism in P. putida KT2440 (19). However, the nature of the regulatory mechanism of glutarate catabolism in P. putida KT2440 and the issue of whether there is an interaction between the processes of regulation of these two pathways have not yet been studied.

In humans, l-2-HG is considered an abnormal metabolite that results in pathogenesis (21–24). It can be produced from the reduction of 2-KG that results from the promiscuous catalytic activity of l-malate dehydrogenase and l-lactate dehydrogenase under acidic and hypoxic conditions (25). In bacteria, l-2-HG is a metabolic intermediate that can be produced from glutarate by CsiD during the catabolism of several organic compounds (such as glutarate, l-lysine, l-tryptophan, and benzoate) (19, 20). l-2-HG is catabolized through the activity of l-2-HG dehydrogenase (L2HGDH) in mammals (26, 27) and l-2-HG oxidase in some bacteria (such as YgaF in E. coli and LhgO in P. putida KT2440) (19, 28). Considering the multiple physiological functions of l-2-HG (29–33), the regulatory mechanism of l-2-HG catabolism also deserves intensive investigation.

In this study, the regulatory mechanism of glutarate catabolism was studied in P. putida KT2440, a model organism containing two glutarate catabolic pathways (19, 34, 35). A GntR family protein, CsiR, and a LysR family protein, GcdR, were identified as the regulators of the glutarate hydroxylation pathway and the glutaryl-CoA dehydrogenation pathway, respectively. There is no cross-regulation between these two pathways. In addition, it was confirmed that CsiR is also involved in l-2-HG catabolism and uses both l-2-HG and glutarate as its effectors. This report improves our understanding of the regulatory mechanisms of glutarate and l-2-HG catabolism.

## RESULTS

### Transcriptional analysis of the genes involved in glutarate catabolism.

**FIG 1:**
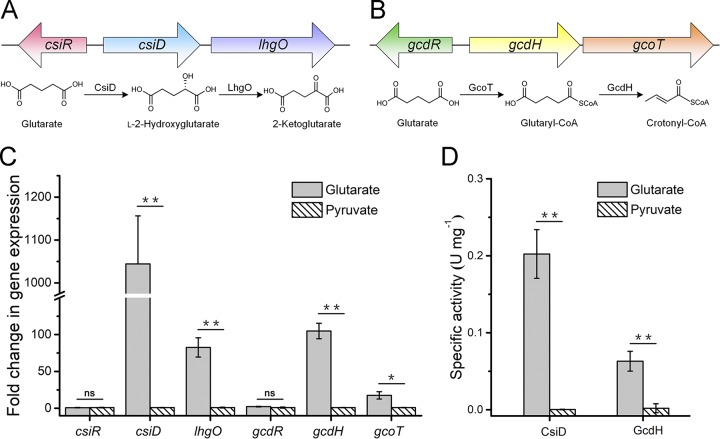
Organization and expression levels of *csiR*-*csiD*-*lhgO* and *gcdR-gcdH-gcoT* gene clusters. (A and B) Schematic representation of the *csiR*-*csiD*-*lhgO* (A) and *gcdR-gcdH-gcoT* (B) gene cluster regions of P. putida KT2440. The corresponding steps of the glutarate degradation pathways are also shown. (C) qPCR analysis of the genes in *csiR*-*csiD*-*lhgO* and *gcdR-gcdH-gcoT* gene clusters. The relative expression levels of six genes, *csiR*, *csiD*, *lhgO*, *gcdR*, *gcdH*, and *gcoT*, were measured using RNA extracted from P. putida KT2440 grown in MSM with glutarate or pyruvate as the sole carbon source. The gene expression levels are represented as expression ratios of the indicated genes in glutarate medium versus pyruvate medium, normalized to 16S rRNA. (D) The activities of CsiD and GcdH in P. putida KT2440 grown in MSM with glutarate or pyruvate as the sole carbon source. Data shown are means ± standard deviations (SD) (*n* = 3 independent experiments). *, *P < *0.05 in two-tailed *t* test; **, *P < *0.01 in two-tailed *t* test; ns, no significant difference (*P *≥* *0.05 in two-tailed *t* test).

Genes *csiD* (P. putida *2909* [*pp2909*]) and *lhgO* (*pp2910*) of the glutarate hydroxylation pathway are adjacent to each other in the genome of P. putida KT2440. A regulator-encoding gene, *csiR* (*pp2908*), can be found upstream of *csiD* (Fig. 1A). There is a *gcdR-gcdH-gcoT* gene cluster that is involved in the glutaryl-CoA dehydrogenation pathway in P. putida KT2440. Gene *gcdH* (*pp0158*) encodes glutaryl-CoA dehydrogenase, while *gcdR* (*pp0157*) and *gcoT* (*pp0159*) encode a LysR family transcriptional regulator (GcdR) and a glutarate-CoA transferase (GcoT), respectively (Fig. 1B).

P. putida KT2440 was cultured in minimal salt medium (MSM) supplemented with glutarate or pyruvate as the sole carbon source, and the transcription levels of the genes mentioned above were detected by reverse transcription-PCR (RT-PCR). Transcription of *csiD* and *lhgO* (involved in the glutarate hydroxylation pathway) was induced by glutarate (19), as was transcription of *gcdH* and *gcoT* (involved in the glutaryl-CoA dehydrogenation pathway) (see Fig. S1A in the supplemental material). The results of the carbon source feeding experiments indicate constitutive expression of genes *csiR* and *gcdR* (Fig. S1B). The relative expression levels of these genes were then analyzed by real-time quantitative PCR (qPCR). As shown in Fig. 1C, genes *csiD*, *lhgO*, *gcdH*, and *gcoT* were induced by glutarate. Additionally, the enzymatic activities of CsiD and GcdH in the glutarate medium were also found to be higher than those in pyruvate medium (Fig. 1D).

10.1128/mBio.01570-19.1 FIG S1 Transcriptional analysis of *gcdH* and *gcoT* (A) and of *csiR* and *gcdR* (B). RT-PCR analyses of mRNAs from P. putida KT2440 cells grown in 5 g liter^−1^ glutarate (lanes 2 and 4) or 5 g liter^−1^ pyruvate (lanes 3 and 5) as the sole carbon source were performed. The reactions were conducted in the presence of a reverse transcriptase (lanes 4 and 5) or in the absence of the enzyme (lanes 2 and 3) (negative control). Genomic DNA was used as a positive control (lane 1). Download FIG S1, TIF file, 0.3 MB. Copyright © 2019 Zhang et al. 2019 Zhang et al. This content is distributed under the terms of the Creative Commons Attribution 4.0 International license.

### CsiR represses the transcription of *csiD* and *lhgO*.

**FIG 2:**
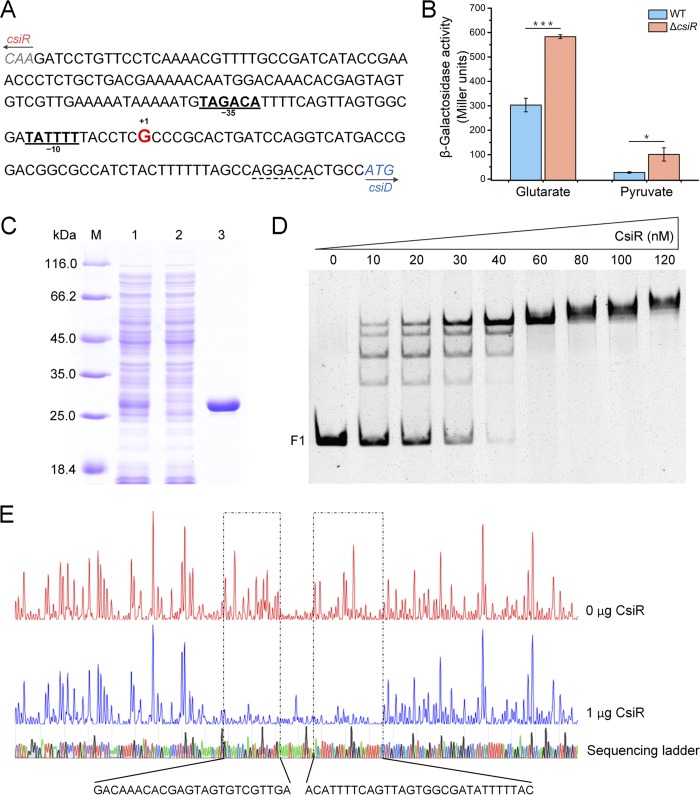
CsiR represses the transcription of *csiD* and *lhgO*. (A) Map of the *csiR*-*csiD* intergenic region. The transcriptional start site (TSS) identified in this study is shown in red letters. The predicted −10 and −35 regions are shown in bold and underlined. The start codons of *csiR* and *csiD* are shown in italics. The ribosome binding site is indicated by dotted lines. (B) The promoter activities of *P_csiD_* in P. putida KT2440 and P. putida KT2440 (Δ*csiR*) cultured in glutarate or pyruvate medium. Data shown are means ± SD (*n *=* *3 independent experiments). (C) SDS-PAGE analysis of steps of expression and purification of CsiR. Lane M, molecular weight markers; lane 1, crude extract of E. coli BL21(DE3) harboring pETDuet-*csiR*; lane 2, the unbound protein of the HisTrap HP column; lane 3, CsiR (purified by the use of a HisTrap column). (D) EMSAs with *csiR*-*csiD* intergenic fragment F1 (10 nM) and purified CsiR (0, 10, 20, 30, 40, 60, 80, 100, and 120 nM). (E) DNase I footprinting analysis of CsiR binding to the *csiD* promoter region. F1 was labeled with FAM dye and incubated with 1 μg CsiR (blue line) or without CsiR (red line). The region protected by CsiR from DNase I cleavage is indicated with a dotted box. *, *P < *0.05 in two-tailed *t* test; ***, *P < *0.001 in two-tailed *t* test.

The transcriptional organization of *csiR*-*csiD*-*lhgO* was then assayed by RT-PCR. The intergenic region of *csiD*-*lhgO* could be amplified whereas the intergenic region of *csiR*-*csiD* could not be amplified (Fig. S2), indicating that genes *csiD* and *lhgO* were cotranscribed but that the transcript corresponding to *csiD* was different from that corresponding to *csiR*. The transcriptional start site (TSS) of the *csiD* gene was determined using the rapid amplification of cDNA ends (RACE) method. The TSS was identified as a guanine (G) residue found 62 bp upstream of the *csiD* start codon, with the putative −10 (TATTTT) and −35 (TAGACA) regions separated by 17 bp (Fig. 2A).

10.1128/mBio.01570-19.2 FIG S2 Transcriptional organization of *csiR*-*csiD*-*lhgO* gene cluster. RT-PCR analyses of mRNAs from P. putida KT2440 cells grown in 5 g liter^−1^ glutarate as sole carbon sources were performed. The reactions were conducted in the presence of a reverse transcriptase (lane 3) or in the absence of the enzyme (lane 2) (negative control). Genomic DNA was used as a positive control (lane 1). Lane M, molecular size marker. Numbers on the left present the sizes of the markers (in base pairs). Download FIG S2, TIF file, 0.2 MB. Copyright © 2019 Zhang et al. 2019 Zhang et al. This content is distributed under the terms of the Creative Commons Attribution 4.0 International license.

To characterize the *csiD* operon promoter, the 134-bp DNA fragment upstream of TSS (G) was fused to *lacZ* of promoter probe plasmid pME6522 to generate pME6522-*P_csiD_* (see Table S1 in the supplemental material). The resulting plasmid was transferred into P. putida KT2440 and P. putida KT2440 (Δ*csiR*) (Table S1) by electroporation, and the promoter activity of *P_csiD_* was measured by β-galactosidase assays after culturing the resulting strains in MSM with glutarate or pyruvate as the sole carbon source. In the presence of glutarate, the promoter activity of *P_csiD_* was significantly higher than that of pyruvate (Fig. 2B), indicating that the *P_csiD_* fragment contains a functional glutarate-responsive promoter. Additionally, the promoter activity of *P_csiD_* in P. putida KT2440 (Δ*csiR*) cultured in glutarate was about twice as high as that of P. putida KT2440, which indicated that CsiR represses the transcription of *csiD*.

10.1128/mBio.01570-19.9 TABLE S1 Strains and vectors used in this study. Download Table S1, DOC file, 0.1 MB. Copyright © 2019 Zhang et al. 2019 Zhang et al. This content is distributed under the terms of the Creative Commons Attribution 4.0 International license.

To determine whether CsiR directly interacts with the *csiD* promoter region, the His_6_-tagged CsiR protein of P. putida KT2440 was expressed in E. coli BL21(DE3) and purified by Ni-chelating chromatography. On the basis of the results of gel filtration and sodium dodecyl sulfate-polyacrylamide gel electrophoresis (SDS-PAGE), CsiR behaved primarily as a dimer (Fig. 2C; see also Fig. S3). Electrophoretic mobility shift assays (EMSAs) were performed using the *csiR*-*csiD* intergenic region DNA fragment (F1) and purified CsiR. As shown in Fig. 2D, CsiR bound to F1 in a concentration-dependent manner and completely shifted the DNA fragment at a 6-fold molar excess. In addition, four DNA-CsiR complexes were detected at low concentrations of CsiR, while the bands shifted to form a more diffuse complex at higher concentrations (>60 nM) of CsiR. These results showed that CsiR can bind to the upstream region of the *csiD* operon.

10.1128/mBio.01570-19.3 FIG S3 Analysis of the native molecular weight of CsiR in P. putida KT2440 by the use of Superdex 200 10/300 GL. Black line, standard curve for protein molecular mass standards; red curve, chromatogram of purified CsiR. Download FIG S3, TIF file, 0.6 MB. Copyright © 2019 Zhang et al. 2019 Zhang et al. This content is distributed under the terms of the Creative Commons Attribution 4.0 International license.

A DNase I footprinting assay was also performed using purified CsiR. The *csiR*-*csiD* intergenic region DNA fragment (F1) end labeled with 6-carboxyfluorescein (FAM) on the noncoding strand was mixed with CsiR protein and was then digested with DNase I. Two clearly protected regions were observed (Fig. 2E). One of the protected regions contained the −10 and −35 regions relative to the TSS of *csiD*, which correlated with the fact that CsiR is a repressor.

### GcdR activates the transcription of *gcdH*.

**FIG 3:**
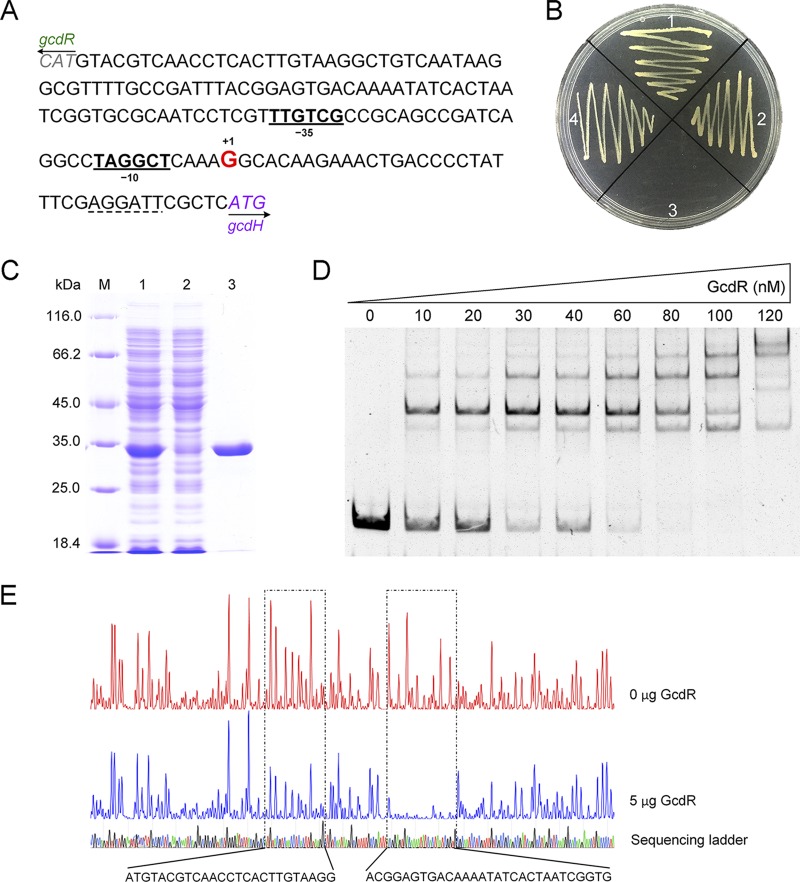
GcdR activates the transcription of *gcdH*. (A) Map of the *gcdR*-*gcdH* intergenic region. The TSS identified in this study is shown in red letters. The predicted −10 and −35 regions are shown in bold and underlined. The start codons of *gcdR* and *gcdH* are shown in italics. The ribosome binding site is indicated by dotted lines. (B) Growth of P. putida KT2440 and its derivatives on solid MSM containing 5 g liter^−1^ glutarate as the sole carbon source. Pictures were taken at 36 h. Section 1, P. putida KT2440; section 2, P. putida KT2440 (Δ*csiD*); section 3, P. putida KT2440 (Δ*csiD* Δ*gcdR*); section 4, P. putida KT2440 (Δ*gcdR*). (C) SDS-PAGE analysis of steps of expression and purification of GcdR. Lane M, molecular weight markers; lane 1, crude extract of E. coli BL21(DE3) harboring pET28a-*gcdR*; lane 2, the unbound protein of the HisTrap HP column; lane 3, purified GcdR using a HisTrap column. (D) EMSAs with the F2 fragment containing the *gcdR*-*gcdH* intergenic region (10 nM) and purified GcdR (0, 10, 20, 30, 40, 60, 80, 100, and 120 nM). (E) DNase I footprinting analysis of GcdR binding to the *gcdH* promoter region. F2 was labeled with FAM dye and incubated with 5 μg GcdR (blue line) or without GcdR (red line). Each region protected by GcdR from DNase I cleavage is indicated with a dotted box.

The transcriptional organization of *gcdR*-*gcdH*-*gcoT* was also assayed by RT-PCR. The intergenic region of *gcdH*-*gcoT* was amplified, while the intergenic region of *gcdR*-*gcdH* could not be detected, indicating that genes *gcdH* and *gcoT* were cotranscribed but that the transcript corresponding to *gcdH* is different from that corresponding to *gcdR* (Fig. S4). The TSS of the *gcdH* operon was a G residue found 37 bp upstream of the *gcdH* start codon, with the putative −10 (TAGGCT) and −35 (TTGTCG) regions separated by 17 bp (Fig. 3A).

10.1128/mBio.01570-19.4 FIG S4 Transcriptional organization of *gcdR*-*gcdH*-*gcoT* gene cluster. RT-PCR analyses of mRNAs from P. putida KT2440 cells grown in 5 g liter^−1^ glutarate as sole carbon sources were performed. The reactions were conducted in the presence of a reverse transcriptase (lane 3) or in the absence of the enzyme (lane 2) (negative control). Genomic DNA was used as a positive control (lane 1). Lane M, molecular size marker. Numbers on the left present the sizes of the markers (in base pairs). Download FIG S4, TIF file, 0.2 MB. Copyright © 2019 Zhang et al. 2019 Zhang et al. This content is distributed under the terms of the Creative Commons Attribution 4.0 International license.

The 124-bp DNA fragment upstream of TSS (G) of *gcdH* was fused to *lacZ* of pME6522 to generate pME6522-*P_gcdH_* (Table S1). The resulting plasmid was transferred into P. putida KT2440 and P. putida KT2440 (Δ*gcdR*) by electroporation, and the promoter activity of *P_gcdH_* was measured by β-galactosidase assays. P. putida KT2440 harboring pME6522-*P_gcdH_* showed a higher β-galactosidase activity under conditions of culturing in the presence of glutarate (Fig. S5), which confirmed that the *P_gcdH_* fragment contains the glutarate-inducible promoter. However, very low activity (<40 Miller units) was detected in P. putida KT2440 (Δ*gcdR*) harboring pME6522-*P_gcdH_* in the presence of glutarate (Fig. S5). The *gcdR* gene in P. putida KT2440 (Δ*csiD*) was then also disrupted to create another mutant strain. The mutant strain, P. putida KT2440 (Δ*csiD*), harboring only the glutaryl-CoA dehydrogenation pathway, was still able to grow on glutarate, while the P. putida KT2440 (Δ*csiD* Δ*gcdR*) strain lost the ability to utilize glutarate (Fig. 3B). These results indicated that GcdR is a transcriptional activator and is indispensable for the transcription of *gcdH*.

10.1128/mBio.01570-19.5 FIG S5 The promoter activity of *P_gcdH_* in P. putida KT2440 and P. putida KT2440 (Δ*gcdR*) cultured in glutarate or pyruvate medium. Data shown are means ± SD (*n *=* *3 independent experiments). **, *P < *0.01 in two-tailed *t* test; ns, no significant difference (*P *≥* *0.05 in two-tailed *t* test). Download FIG S5, TIF file, 0.3 MB. Copyright © 2019 Zhang et al. 2019 Zhang et al. This content is distributed under the terms of the Creative Commons Attribution 4.0 International license.

The His_6_-tagged GcdR of P. putida KT2440 was expressed in E. coli BL21(DE3) and purified. On the basis of the results of gel filtration and SDS-PAGE, GcdR behaved primarily as a tetramer (Fig. 3C; see also Fig. S6). GcdR could not completely bind to the *gcdR*-*gcdH* intergenic region in EMSAs. The intergenic region was extended by adding 50 bp both upstream and downstream (the resulting region was named F2). GcdR bound to F2 in a concentration-dependent manner and completely shifted the DNA fragment at 10-fold molar excess (Fig. 3D). A DNase I footprinting assay was then performed using purified GcdR and F2. Two clearly protected regions were determined (Fig. 3E). One of the protected regions contained the **T**-N_11_-**A** consensus binding motif of LysR-type transcriptional regulators (36, 37), 5′-G**T**GACAAAATATC**A**C-3′, and an interrupted inverted repeat sequence, 5′-AG**T**GA-N_7_-TC**A**CT-3′ (the start and end of the relevant sequences are indicated in bold).

10.1128/mBio.01570-19.6 FIG S6 Analysis of the native molecular weight of GcdR in P. putida KT2440 by the use of Superdex 200 10/300 GL. Black line, standard curve for protein molecular mass standards; red curve, chromatogram of purified GcdR. Download FIG S6, TIF file, 0.4 MB. Copyright © 2019 Zhang et al. 2019 Zhang et al. This content is distributed under the terms of the Creative Commons Attribution 4.0 International license.

### CsiR and GcdR regulate their own target pathways.

**FIG 4:**
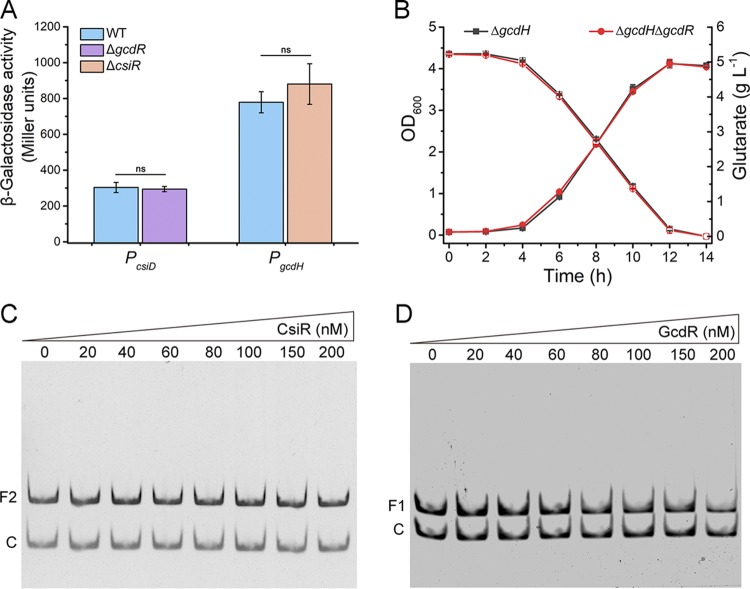
CsiR and GcdR regulate their own target pathways. (A) The promoter activities of *P_csiD_* and *P_gcdH_* in P. putida KT2440, P. putida KT2440 (Δ*gcdR*) (for the determination of *P_csiD_* data), and P. putida KT2440 (Δ*csiR*) (for the determination of *P_gcdH_* data). (B) Growth of P. putida KT2440 (Δ*gcdH*) and P. putida KT2440 (Δ*gcdH* Δ*gcdR*) in MSM with glutarate as the sole carbon source. The levels of growth (closed symbols) and of consumption of glutarate (open symbols) were measured. Data shown are means ± SD (*n *=* *3 independent experiments). (C) EMSAs with F2 (10 nM) and purified CsiR (0, 20, 40, 60, 80, 60, 100, 150, and 200 nM). (D) EMSAs with F1 (10 nM) and purified GcdR (0, 20, 40, 60, 80, 60, 100, 150, and 200 nM). A 148-bp internal fragment of *csiD* (10 nM) was used as a negative control (C rows). ns, no significant difference (*P *≥* *0.05 in two-tailed *t* test).

In order to determine whether there was cross-regulation between CsiR and GcdR, we used *lacZ* transcriptional fusions to measure the impact of *csiR* and *gcdR* mutants on the expression of the *gcdH* and *csiD* genes, respectively. As shown in Fig. 4A, the disruption of *gcdR* had no effect on the activity of the *P_csiD_* promoter, and the disruption of *csiR* had no effect on the activity of the *P_gcdH_* promoter too. It was shown that the regulator of one glutarate metabolic pathway could not activate or inhibit the transcription of genes in the other pathway, indicating that the two pathways are likely to be independent of each other and that there is no cross-regulation.

The *gcdR* gene in P. putida KT2440 (Δ*gcdH*) was then also disrupted. P. putida KT2440 (Δ*gcdH*) harbored only the glutarate hydroxylation pathway. The levels of growth and glutarate consumption of P. putida KT2440 (Δ*gcdH* Δ*gcdR*) were the same as those seen with P. putida KT2440 (Δ*gcdH*) (Fig. 4B), further suggesting that GcdR may not regulate the glutarate hydroxylation pathway. Results of EMSAs also indicated that CsiR could not directly interact with the *gcdH* promoter region (Fig. 4C) and that GcdR could not directly interact with the *csiD* promoter region (Fig. 4D). Taken together, these results suggested that CsiR and GcdR regulate their own target pathways.

### Characterization of the effectors of CsiR and GcdR.

**FIG 5:**
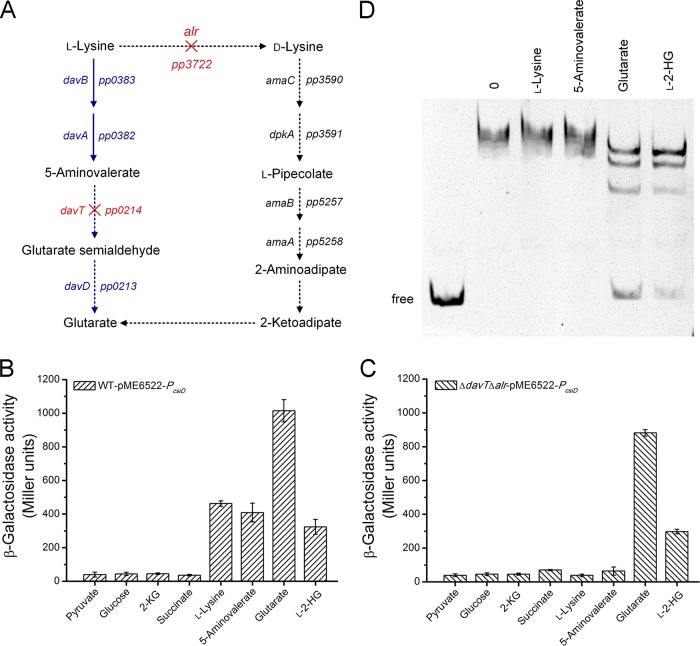
Characterization of the effectors of CsiR. (A) Schematic representation of l-lysine catabolism in P. putida KT2440 and the influences of *davT* and *alr* deletions. Pathways whose activity could not continue after the deletions of *davT* and *alr* are indicated by solid dashed arrows. *davB*, l-lysine monooxygenase; *davA*, 5-aminovaleramide amidohydrolase; *davT*, 5-aminovalerate aminotransferase; *davD*, glutaric semialdehyde dehydrogenase; *alr*, alanine racemase; *amaC*, d-lysine aminotransferase; *dpkA*, Δ^1^-piperideine-2-carboxylate reductase; *amaB*, l-pipecolate oxidase; *amaA*, l-piperidine-6-carboxylate dehydrogenase. (B and C) The β-galactosidase assays were performed with P. putida KT2440-pME6522-*P_csiD_* (B) and P. putida KT2440 (Δ*davT* Δ*alr*)-pME6522-*P_csiD_* (C) grown in MSMs with 2.5 g liter^−1^ pyruvate and different compounds as the carbon sources. Data shown are means ± SD (*n *=* *3 independent experiments). (D) Glutarate and l-2-HG prevent CsiR binding to F1. EMSAs were performed with F1 (10 nM) and a 5-fold molar excess of CsiR in the absence of any other tested compounds (0) and in the presence of 40 mM l-lysine, 5-aminovalerate, glutarate, and l-2-HG. The leftmost lane shows the migration of free DNA (no CsiR).

The effects of glutarate and other compounds involved in the glutarate metabolism on the activity of the *csiD* promoter were evaluated. Plasmid pME6522-*P_csiD_* was transferred into P. putida KT2440 (Δ*davT* Δ*alr*) by electroporation, and the resulting strain was incapable of converting l-lysine and 5-aminovalerate into glutarate (Fig. 5A). The promoter activities of *P_csiD_* were measured using β-galactosidase assays, and the wild-type strain and P. putida KT2440 (Δ*davT* Δ*alr*) harboring pME6522-*P_csiD_* were cultured in MSMs with 2.5 g liter^−1^ pyruvate and different compounds as the carbon sources. Significant levels (324 to 1,015 Miller units) of promoter activities were detected in P. putida KT2440 harboring pME6522-*P_csiD_* in the presence of l-lysine, 5-aminovalerate, glutarate, and l-2-HG (Fig. 5B), whereas the β-galactosidase activities were observed only when P. putida KT2440 (Δ*davT* Δ*alr*) harboring pME6522-*P_csiD_* was grown in the presence of glutarate and l-2-HG (Fig. 5C). These results suggested that glutarate and l-2-HG can induce the *P_csiD_* promoter whereas l-lysine and 5-aminovalerate cannot induce the *P_csiD_* promoter.

The effects of l-lysine, 5-aminovalerate, glutarate, and l-2-HG on CsiR binding to the *csiD* promoter region were assessed by EMSAs. l-Lysine and 5-aminovalerate had no effect on the binding of CsiR to F1, whereas glutarate and l-2-HG prevented the binding (Fig. 5D). The effects of glutarate and l-2-HG (10 mM, 20 mM, 40 mM, 60 mM, 80 mM, 100 mM, and 120 mM) on the capacity of binding of CsiR to F1 were further analyzed by EMSAs. When the concentrations of glutarate and l-2-HG were increased, the amount of the CsiR-DNA complex decreased and the amount of the free DNA increased (Fig. S7). Therefore, glutarate and l-2-HG are the effectors of CsiR.

10.1128/mBio.01570-19.7 FIG S7 The influence of the concentrations of glutarate (A) and l-2-HG (B) on the binding of CsiR to DNA. EMSAs were performed with F1 (10 nM) in the presence of increasing amounts of glutarate and l-2-HG (0 to 120 mM). The leftmost lane indicates the migration of free DNA. The other lanes contain F1 and CsiR and increasing concentrations of the tested compounds (as indicated). Download FIG S7, TIF file, 1.8 MB. Copyright © 2019 Zhang et al. 2019 Zhang et al. This content is distributed under the terms of the Creative Commons Attribution 4.0 International license.

**FIG 6:**
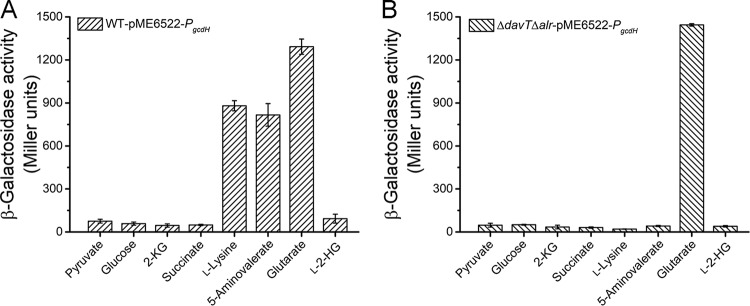
Characterization of the effector of GcdR. The β-galactosidase assays were performed with P. putida KT2440-pME6522-*P_gcdH_* (A) and P. putida KT2440 (Δ*davT* Δ*alr*)-pME6522-*P_gcdH_* (B) grown in MSMs with 2.5 g liter^−1^ pyruvate and different compounds as the carbon sources. Data shown are means ± SD (*n *=* *3 independent experiments).

As for GcdR, l-lysine, 5-aminovalerate, and glutarate could induce the *gcdH* promoter in P. putida KT2440 harboring pME6522-*P_gcdH_* (Fig. 6A). However, only glutarate could induce the *gcdH* promoter in P. putida KT2440 (Δ*davT* Δ*alr*) harboring pME6522-*P_gcdH_*, while l-lysine and 5-aminovalerate could not (Fig. 6B). Thus, glutarate is the effector of GcdR.

### CsiR regulates the catabolism of l-2-HG.

**FIG 7:**
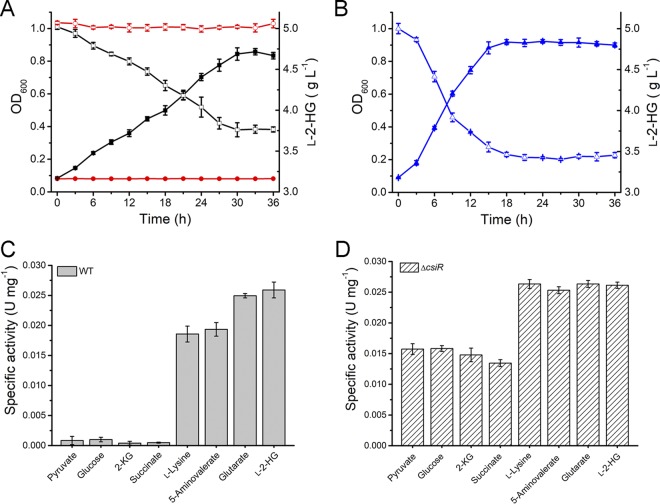
CsiR regulates the catabolism of l-2-HG. (A) Growth of P. putida KT2440 and its *lhgO* mutant in MSM with l-2-HG as the sole carbon source. Growth (closed symbols) and the consumption of l-2-HG (open symbols) of wild-type P. putida KT2440 (black lines with squares) and its *lhgO* mutant (red lines with circles) were measured in MSM supplemented with 5 g liter^−1^ l-2-HG as the sole carbon source. (B) Growth (closed symbols) and consumption of l-2-HG (open symbols) of P. putida KT2440 (Δ*csiR*) in MSM with l-2-HG as the sole carbon source. (C) The activity of LhgO in P. putida KT2440 cultured in MSMs with different compounds as the sole carbon sources. (D) The activity of LhgO in P. putida KT2440 (Δ*csiR*) cultured in MSMs with different compounds as the sole carbon sources. Data shown are means ± SD (*n *=* *3 independent experiments).

As CsiR was able to use both glutarate and l-2-HG as the effectors, it was speculated that CsiR also regulates the metabolism of l-2-HG. Although P. putida KT2440 was able to use l-2-HG as the sole carbon source, it lost the ability to utilize l-2-HG after the *lhgO* gene was deleted (Fig. 7A), suggesting that *lhgO* was indispensable for l-2-HG utilization. The disruption of *csiR* significantly increased the growth rate and l-2-HG consumption rate of P. putida KT2440 in MSM with l-2-HG as the sole carbon source (Fig. 7B), implying that CsiR may repress the utilization of l-2-HG.

The activity of LhgO in P. putida KT2440 was detected using the wild-type strain and P. putida KT2440 (Δ*csiR*) cultured in media with different compounds as the sole carbon sources. l-2-HG, l-lysine, 5-aminovalerate, and glutarate were found to induce the expression of LhgO, whereas 2-KG, glucose, succinate, and pyruvate did not (Fig. 7C). Moreover, the enzymatic activity of LhgO in P. putida KT2440 (Δ*csiR*) was detected in the media with all of the compounds described above as the sole carbon sources (Fig. 7D).

The growth rate and l-2-HG consumption rate of P. putida KT2440 (Δ*gcdR*) were consistent with those of the wild-type strain when l-2-HG was used as the sole carbon source (Fig. S8A). When P. putida KT2440 (Δ*gcdR*) was cultured in MSM with different compounds as the sole carbon sources, the activity of LhgO in all the tested compounds was consistent with that of the wild-type strain (Fig. S8B). These results indicate that CsiR regulates the catabolism of l-2-HG, while GcdR does not.

10.1128/mBio.01570-19.8 FIG S8 GcdR was unable to regulate the expression of *lhgO* gene. (A) Growth (closed symbols) and consumption of l-2-HG (open symbols) of P. putida KT2440 (Δ*gcdR*) in MSM with l-2-HG as the sole carbon source. (B) The activity of LhgO in P. putida KT2440 (Δ*gcdR*) cultured in MSMs with different compounds as the sole carbon sources. Data shown are means ± SD (*n *=* *3 independent experiments). Download FIG S8, TIF file, 0.3 MB. Copyright © 2019 Zhang et al. 2019 Zhang et al. This content is distributed under the terms of the Creative Commons Attribution 4.0 International license.

## DISCUSSION

**FIG 8:**
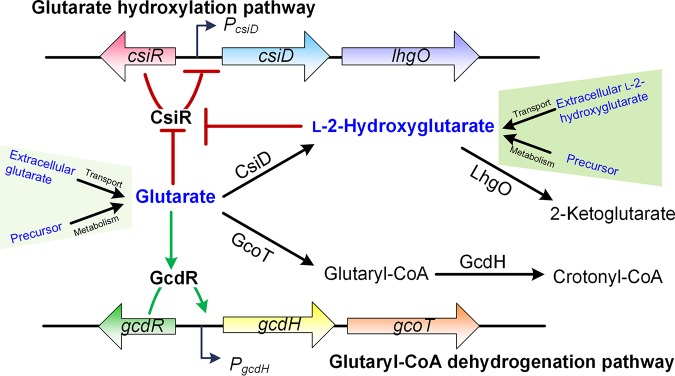
The proposed model for the regulation of glutarate catabolism by CsiR and GcdR in P. putida KT2440. The CsiR regulator represses the expression of *csiD*-*lhgO* genes in the glutarate hydroxylation pathway. Glutarate and l-2-HG from metabolism of their respective precursors or extracellular transport are effectors of CsiR and prevent CsiR binding to the *csiD* promoter region (red arrows). The GcdR regulator is activated by glutarate, thereby initiating expression of *gcdH*-*gcoT* genes in the glutaryl-CoA dehydrogenation pathway (green arrows).

On the basis of the results described above, we proposed a model of the regulation of glutarate and l-2-HG catabolism in P. putida KT2440 (Fig. 8). The catabolism of glutarate is regulated by CsiR and GcdR, which control the glutarate hydroxylation pathway and glutaryl-CoA dehydrogenation pathway, respectively. CsiR is a transcriptional regulator in the GntR family and can specifically bind to the *csiD* promoter region and repress the transcription of the *csiD* and *lhgO* genes. GcdR is a transcriptional regulator in the LysR family and can specifically bind to the *gcdH* promoter region and activate the transcription of *gcdH*.

The catabolism of glutarate in P. aeruginosa PAO1 depends on GcdH, whose expression is under the control of the GcdR transcriptional activator (38). The catabolism of glutarate in E. coli depends on CsiD and LhgO (20). The expression of *csiD* in E. coli is significantly upregulated during carbon starvation (39). However, the two pathways cooperate in glutarate catabolism in P. putida KT2440 and both GcdH and CsiD are induced during carbon starvation (19). In this study, it was found that two regulators, CsiR and GcdR, control the two pathways described above in P. putida KT2440, respectively. CsiR cannot interact with the *gcdH* promoter region (Fig. 4C) and has no effect on the transcription of *gcdH* (Fig. 4A). Similarly, GcdR cannot interact with the *csiD* promoter region (Fig. 4D) and has no effect on the transcription of *csiD* (Fig. 4A). In contrast to GcdH, which is present universally in *Pseudomonas* species, CsiD and LhgO may be acquired via horizontal gene transfer and are sporadically distributed in only 25 strains of *Pseudomonas* (19). The independence of the processes of evolution of the two pathways may be the cause for their independent regulation. However, the absence of cross-regulation between the two pathways does not eliminate the possibility that CsiR and GcdR can also regulate other genes in addition to their respective targets in glutarate catabolism. The potential alternative binding sites of CsiR and GcdR might be identifiable through transcriptome-based bioinformatic analysis and successive experimental validation.

GcdR uses only glutarate, the substrate of the glutaryl-CoA dehydrogenation pathway, as its effector. However, CsiR in P. putida KT2440 uses two effectors, the substrate glutarate and the intermediate l-2-HG of the glutarate hydroxylation pathway (Fig. 8). The effector of the transcriptional repressor protein is usually the substrate of the operon or a specific intermediate in the metabolism of the substrate. For instance, the effector of the l-lactate operon in E. coli is the substrate l-lactate (40). The effector of the 2,3-butanediol operon in P. aeruginosa PAO1 is the metabolic intermediate acetaldehyde (41). Although the effector promiscuity of CsiR may be due to the structural similarity of l-2-HG and glutarate, it is also possible that the response of CsiR to l-2-HG has a physiological significance.

Interestingly, P. putida KT2440 can use l-2-HG as the sole carbon source for growth and the utilization of l-2-HG depends on *lhgO*, a gene in the *csiD*-*lhgO* operon regulated by CsiR (Fig. 7A). l-2-HG also has some important physiological functions, including helping cells adapt to hypoxia, enhancing immunity, and metabolizing several compounds (19, 29–32). In addition to a metabolic intermediate that can be produced during the glutarate metabolism, l-2-HG can also be produced from the reduction of 2-KG by the promiscuous catalytic activity of l-malate dehydrogenase and l-lactate dehydrogenase (25). The excessive accumulation of l-2-HG can be toxic to cells (22, 42, 43). Under conditions where l-2-HG but not glutarate is present, using l-2-HG as the effector of CsiR could help P. putida KT2440 to quickly sense l-2-HG present in habitats or produced by the intracellular metabolism to regulate the utilization of l-2-HG. CsiR is the first regulator that has been identified as being involved in the l-2-HG catabolism and that uses l-2-HG as its effector. This finding could be helpful in the identification of the regulatory proteins of l-2-HG catabolism in other species.

Glutarate is a five-carbon dicarboxylic acid with important industrial applications (1, 2). In recent years, increased attention has been paid to the biotechnological production of glutarate. For example, glutarate can be produced by the four-step degradation of l-lysine (1, 2, 8, 44). In addition, glutarate can be produced through the reverse adipate degradation pathway or α-keto acid carbon chain extension pathway (3, 10). The elucidation of the regulatory mechanism of the glutarate metabolism could help to block the glutarate metabolic pathway, thereby improving glutarate production through biotechnological routes. Glutaric aciduria type I is an inherited metabolic disorder (45, 46). In most cases, the diagnosis of glutaric aciduria type I is established biochemically by the detection of glutarate (47, 48). The elucidation of the regulatory response to glutarate could also help to develop glutarate biosensors and a rapid detection method for glutarate. Recently, both CsiR and GcdR have been used in the development of glutarate biosensors (49). In this study, we confirmed that CsiR uses both glutarate and l-2-HG as effectors. Thus, GcdR may be a more suitable regulatory protein for use in construction of these biosensors.

In summary, we have demonstrated that the catabolism of glutarate in P. putida KT2440 is regulated by the glutarate hydroxylation pathway regulator, CsiR, and by the glutaryl-CoA dehydrogenation pathway regulator, GcdR. The two metabolic pathways are independent of each other in regulation. CsiR is a special transcriptional repressor with two effectors, i.e., glutarate and l-2-HG. It is also the first regulatory protein to be identified in the regulation of the catabolism of l-2-HG.

## MATERIALS AND METHODS

### Bacterial strains and culture conditions.

The bacterial strains and plasmids used in this study are listed in Table S1 in the supplemental material. E. coli was grown in Luria-Bertani (LB) medium at 37°C. P. putida KT2440 and its derivatives were cultivated in minimal salt medium (MSM) supplemented with different compounds as the sole carbon source at 30°C. If necessary, antibiotics were used at the following concentrations: kanamycin, 50 μg ml^−1^; ampicillin, 100 μg ml^−1^; and tetracycline, 30 μg ml^−1^. Cell growth was monitored by measuring turbidity at 600 nm.

### RT-PCR and qPCR.

P. putida KT2440 and its derivatives were cultivated in MSM supplemented with the appropriate carbon sources. Total bacterial RNA was purified by the use of an RNAprep Pure cell/bacteria kit (Tiangen Biotech, China) according to the manufacturer’s directions. Contaminating DNA in the RNA preparations was removed by the use of RNase-free DNase I (TransGen, China). Synthesis of cDNA was performed using Superscript II reverse transcriptase (TransGen, China).

For transcriptional and cotranscriptional analysis, RT-PCR analyses were performed using mRNAs of P. putida KT2440 cells cultured in MSM and the appropriate primers. RT-qPCR was performed by the use of TransStart Top Green qPCR SuperMix (TransGen, China) and a LightCycler 480 system (Roche). The relative levels of expression of the genes were calculated using the threshold cycle (2^−ΔΔ^*^CT^*) method (50). The results were normalized to the 16S rRNA gene level.

### Enzymatic assays of CsiD, GcdH, and LhgO.

P. putida KT2440 was grown to mid-log stage in MSMs supplemented with the appropriate compounds as the sole carbon sources at 200 rpm and 30°C. Cells were harvested, centrifuged, washed, and resuspended in phosphate-buffered saline (PBS). Cells were adjusted to a final optical density at 600 nm (OD_600_) of 20 and sonicated with a Sonics sonicator (500 W, 20 KHz). The homogenate was centrifuged at 13,000 × *g* for 5 min at 4°C, and the supernatants were used as the crude cell extracts for activity measurement.

The activity of CsiD was measured at 30°C in 500 μl of a reaction solution containing 20 mM imidazole (pH 6.7), 1 mM glutarate, 1 mM 2-KG, 0.4 mM ascorbate, 50 μM Fe^2+^, and 40 μl crude cell extracts. The consumption of oxygen was measured using a Clark-type oxygen electrode (Oxytherm; Hansatech, United Kingdom) equipped with an automatically temperature-controlled electrode chamber. One unit (U) of CsiD activity was defined as the amount that catalyzed the reduction of 1 μmol of oxygen per min.

The activity of GcdH was determined at 30°C in 200 μl of a reaction solution containing 50 mM Tris-HCl (pH 7.4), 0.25 mM glutaryl-CoA, 0.01 μM flavin adenine dinucleotide (FAD), 0.2 mM ferricenium hexafluorophosphate (51), and 30 μl crude cell extracts. The absorbance at 300 nm was measured using a Spectramax Plus 384 spectrophotometer (Molecular Devices, USA). One unit (U) of GcdH activity was defined as the amount that catalyzed the reduction of 1 μmol of ferricenium hexafluorophosphate per min.

The activity of LhgO was measured at 30°C in 800 μl of a reaction solution containing PBS, 0.1 mM l-2-HG, 0.05 mM dichlorophenol-indophenol (DCPIP), 0.2 mM phenazine methosulfate (PMS), and 40 μl crude cell extracts. The absorbance at 600 nm was measured using a UV/visible light spectrophotometer (Ultrospec 2100 pro; Amersham Biosciences, USA). One unit (U) of LhgO activity was defined as the amount that catalyzed the reduction of 1 μmol of DCPIP per min.

### Construction of P. putida KT2440 mutants.

The P. putida KT2440 (Δ*csiR*) mutant was generated as follows: the homologous arms upstream and downstream of the *csiR* gene were PCR amplified using primer pair *csiR*-uf/*csiR*-ur and primer pair *csiR*-df/*csiR*-dr, respectively (Table S2). The upstream and downstream fragments were fused via recombinant PCR with primers *csiR*-uf and *csiR*-dr. The generated fusion was digested with BamHI and HindIII and cloned into pK18*mobsacB* (52) cut with the same enzymes. The resulting plasmid, pK18*mobsacB*-Δ*csiR*, was transferred into P. putida KT2440 by electroporation, and the mutant with integration of the plasmid pK18*mobsacB*-Δ*csiR* into the chromosome was obtained by selection on an LB plate containing 50 μg ml^−1^ kanamycin. Kanamycin-resistant transformants were plated onto LB plates containing 10% (wt/vol) sucrose to screen the *csiR* deletion mutants. All the constructed strains were confirmed by PCR and sequence analysis. Other mutants of P. putida KT2440 were generated using the same procedure.

10.1128/mBio.01570-19.10 TABLE S2 Primers used in this study. Download Table S2, DOC file, 0.10 MB. Copyright © 2019 Zhang et al. 2019 Zhang et al. This content is distributed under the terms of the Creative Commons Attribution 4.0 International license.

### Expression and purification of recombinant CsiR and GcdR.

The *csiR* and *gcdR* genes were amplified using genomic DNA of P. putida KT2440 and primer pair *csiR*-F/*csiR*-R and primer pair *gcdR*-F/*gcdR*-R, respectively (Table S2). The amplified *csiR* gene was cut with BamHI and HindIII and cloned into His tag expression vector pETDuet-1 to generate pETDuet-*csiR*. The amplified *gcdR* gene was digested with NcoI and HindIII and cloned into His tag expression vector pET28a to generate pET28a-*gcdR.* The resulting expression plasmids were transformed into E. coli BL21(DE3) for CsiR and GcdR expression.

The recombinant E. coli BL21(DE3) strains containing either plasmid pETDuet-*csiR* or plasmid pET28a-*gcdR* were grown at 37°C in LB medium to an OD_600_ of 0.5 to 0.6 and induced at 16°C for 10 h in the presence of 1 mM isopropyl-d-1-thiogalactopyranoside (IPTG). The cells were collected by centrifugation and washed twice with buffer A (20 mM sodium phosphate and 500 mM sodium chloride, pH 7.4). Pellets were resuspended in buffer A containing 1 mM phenylmethylsulfonyl fluoride (PMSF) and 10% glycerol (vol/vol) and were then lysed by sonication. The cellular lysate was centrifuged at 16,000 × *g* for 30 min at 4°C to remove bacterial debris. The supernatant was loaded onto a HisTrap HP column (5 ml) that was preequilibrated with buffer A. Proteins were eluted with buffer B (20 mM sodium phosphate, 500 mM imidazole, and 500 mM sodium chloride, pH 7.4), and the eluted fractions were analyzed by sodium dodecyl sulfate-polyacrylamide gel electrophoresis (SDS-PAGE) with 12.5% polyacrylamide gels. Protein concentrations were determined by the use of Bradford assays.

The native molecular weights of CsiR and GcdR in P. putida KT2440 were determined using a gel filtration column (Superdex 200 10/300 GL; GE Healthcare). The eluent buffers used for determination of CsiR and GcdR data were buffer C (50 mM sodium phosphate and 150 mM sodium chloride, pH 7.2) and buffer A, respectively. The flow rate was 0.5 ml min^−1^ throughout. Thyroglobulin (669 kDa), ferritin (440 kDa), aldolase (158 kDa), conalbumin (75 kDa), ovalbumin (43 kDa), and RNase A (13.7 kDa) were used as standard proteins.

### Determination of the transcriptional start sites.

RNA samples were isolated from P. putida KT2440 grown in MSM supplemented with glutarate as the sole carbon source. The transcriptional start sites of the *csiD* and *gcdH* operons were determined using a 5′ rapid amplification of cDNA ends (RACE) system (Invitrogen, China). As for *csiD*, the first strand cDNA was synthesized from total RNA using primer *csiD*-GSP1 (Table S2). The resulting cDNA was tailed with terminal deoxynucleotidyl transferase and dCTP and was subsequently amplified via PCR with the abridged anchor primer (APP) and *csiD*-GSP2 (Table S2). A nested PCR was then performed using the PCR product as a template with AAP and *csiD*-GSP3 (Table S2). The resulting PCR product was cloned into pMD18-T vector (TaKaRa, China) for sequencing. The transcriptional start site of the *gcdH* operon was determined using the same procedure.

### Electrophoretic mobility shift assays.

The DNA fragments used in the EMSAs were obtained from P. putida KT2440 genomic DNA by PCR using primers F1-F/F1-R, primers F2-F/F2-R, and primers C-F/C-R (Table S2). EMSAs were carried out using 20-μl reaction mixtures containing 10 nM DNA fragment and increasing concentrations (0 to 120 nM) of purified proteins in EMSA binding buffer (10 mM Tris-HCl [pH 7.4], 50 mM KCl, 0.5 mM EDTA, 10% glycerol, 1 mM dithiothreitol [DTT]). All binding reaction mixtures were incubated at 30°C for 30 min and then subjected to electrophoresis on 6% native polyacrylamide gels for approximately 50 min at 4°C and 170 V (constant voltage). The gels were stained with SYBR green I (TaKaRa, China) for 30 min and photographed under UV irradiation.

To analyze the possible effectors of CsiR, the protein was incubated with l-lysine, 5-aminovalerate, glutarate, or l-2-HG in EMSA buffer at 30°C for 15 min. The F1 DNA fragment (10 nM) was then added, and the reaction mixture was incubated for an additional 30 min before electrophoresis.

### DNase I footprinting.

For preparation of the probes, the fragments containing the intergenic *csiR*-*csiD* region or the *gcdR*-*gcdH* region were amplified by PCR using the appropriate primers (primers F1-F/F1-R and primers F2-F/F2-R) (Table S2). The PCR products were cloned into pEASY-Blunt Simple Cloning Vector (TransGen, China), generating pEASY-Blunt-F1 and pEASY-Blunt-F2, respectively. The probes were obtained by PCR amplification using primers M13F-FAM and M13R and plasmid pEASY-Blunt-F1 or pEASY-Blunt-F2 as the template. Following gel purification, the FAM-labeled probes were quantified with a NanoDrop 2000 spectrophotometer (Thermo Scientific, USA).

For each assay, a 400-ng volume of probe was incubated with CsiR (for the probe containing F1) or GcdR (for the probe containing F2) in a total volume of 40 μl in EMSA binding buffer. After incubation at 30°C for 30 min, all reaction mixtures were treated with 10 μl of a solution containing about 0.015 U DNase I (Promega) and 100 nmol freshly prepared CaCl_2_ and were further incubated at 25°C for 1 min. The reaction was terminated by addition of 140 μl of a stop solution containing 30 mM EDTA, 200 mM sodium acetate, and 0.15% (wt/vol) SDS. Digested samples were extracted with phenol-chloroform, precipitated with ethanol, resuspended in 30 μl MiniQ water, and analyzed as described before (53).

### β-Galactosidase assays.

To construct the reporter plasmids of the *csiD* promoter, a 134-bp fragment upstream of the TSS (*P_csiD_*) was PCR amplified from P. putida KT2440 genomic DNA using the appropriate oligonucleotide pairs (Table S2). The purified PCR products were digested with EcoRI and PstI and cloned into pME6522 to generate pME6522-*P_csiD_*. In the same way, a 124-bp *P_gcdH_* fragment was cloned into pME6522 to generate pME6522-*P_gcdH_*. The resulting plasmids were verified by DNA sequencing and subsequently transferred into P. putida KT2440 and its derivatives by electroporation.

P. putida KT2440 and its derivatives harboring pME6522-*P_csiD_* or pME6522-*P_gcdH_* were grown in MSMs with different compounds as sole carbon sources. Cells were obtained from cultures at the mid-log phase and permeabilized with chloroform and SDS. The β-galactosidase activity was determined using *o*-nitrophenyl-β-d-galactopyranoside as the substrate, and the results were expressed in Miller units (54).

### Quantification of l-2-HG.

P. putida KT2440 and its derivatives were cultured at 200 rpm and 30°C in MSM supplemented with 5.0 g liter^−1^ l-2-HG as the sole carbon source. Samples (1.0 ml) were taken periodically, boiled at 100°C for 15 min, and then centrifuged at 12,000 × *g* for 15 min to remove cell debris. The concentrations of l-2-HG were measured by the use of a high-performance liquid chromatography (HPLC) system (Agilent 1100 series) equipped with an Aminex HPX-87H column (Bio-Rad) and a refractive index detector (RID). The mobile phase was 0.1% formic acid at a flow rate of 0.4 ml min^−1^.
