# Supplementary figures and images for: Regulation of Glutarate Catabolism by GntR Family Regulator CsiR and LysR Family Regulator GcdR in Pseudomonas putida KT2440
Source: mBio. 2019 Jul 30;10(4):e01570-19. doi: 10.1128/mBio.01570-19 (PMC6667623; doi:10.1128/mBio.01570-19)

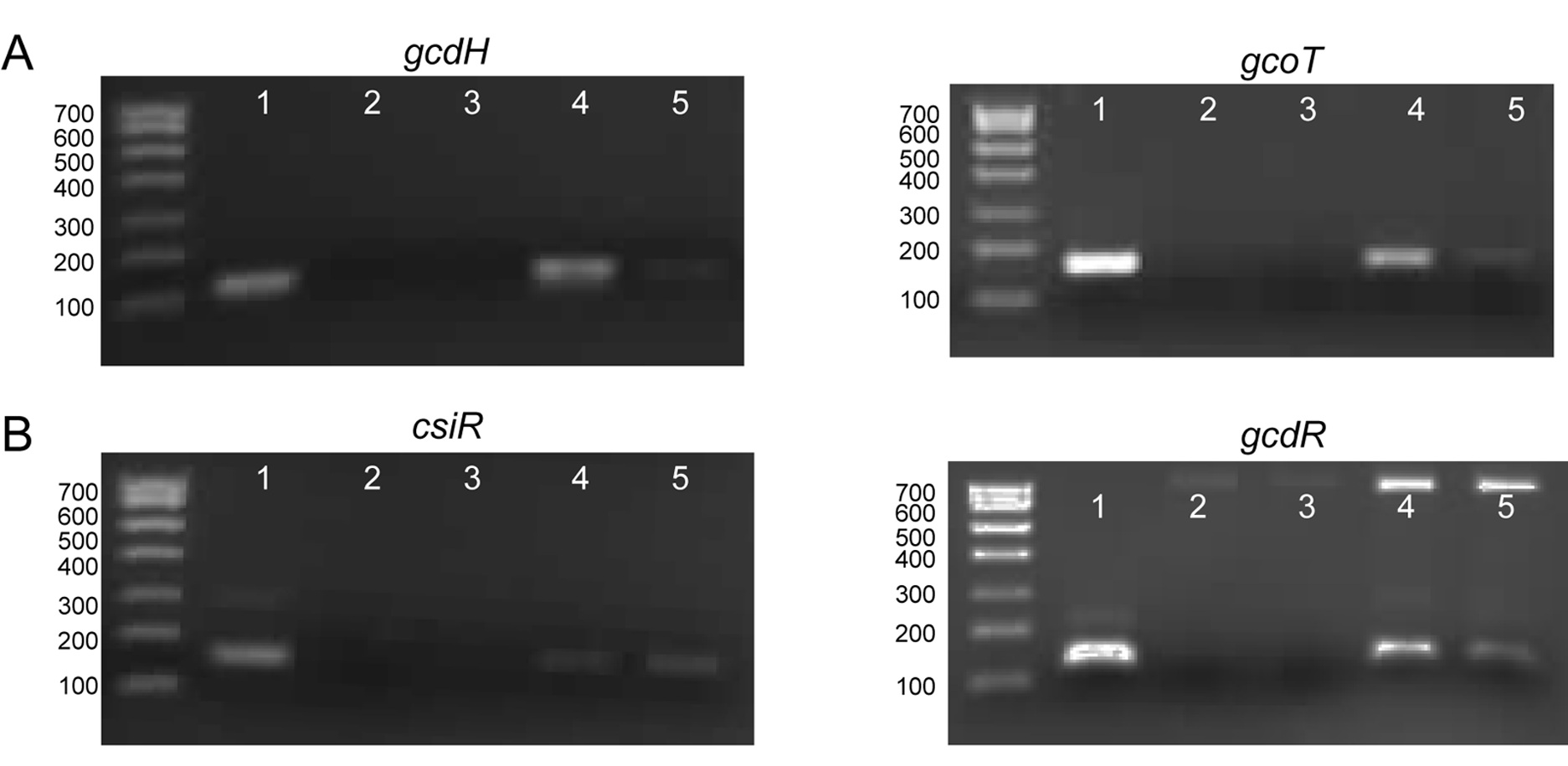

Supplement: FIG S1 [file mBio.01570-19-sf001.tif]

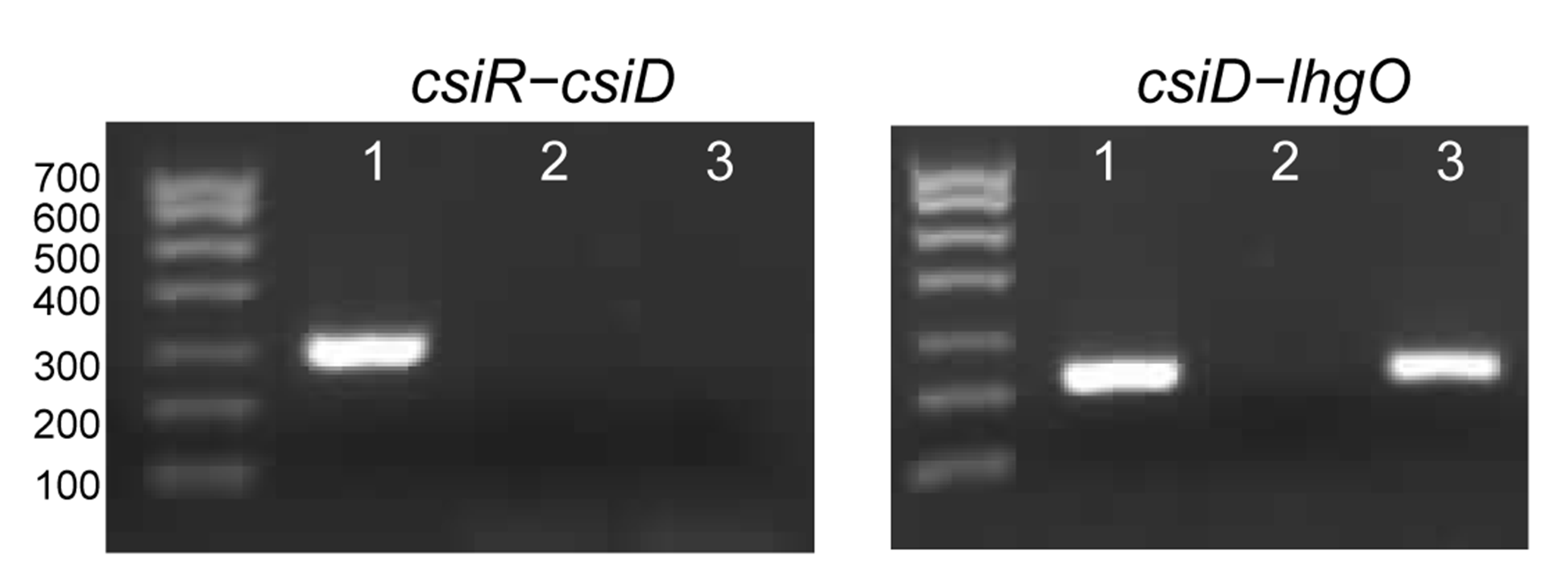

Supplement: FIG S2 [file mBio.01570-19-sf002.tif]

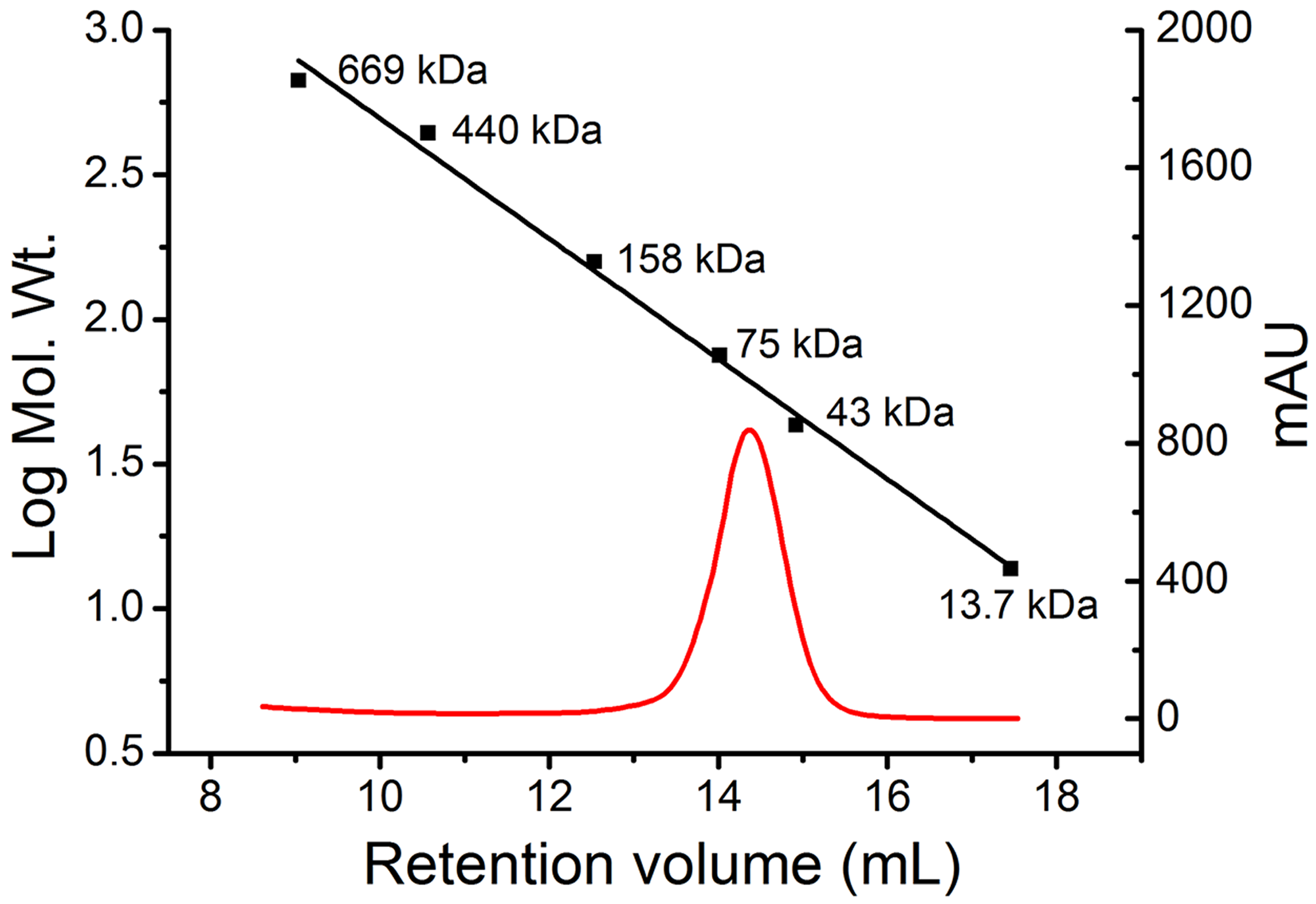

Supplement: FIG S3 [file mBio.01570-19-sf003.tif]

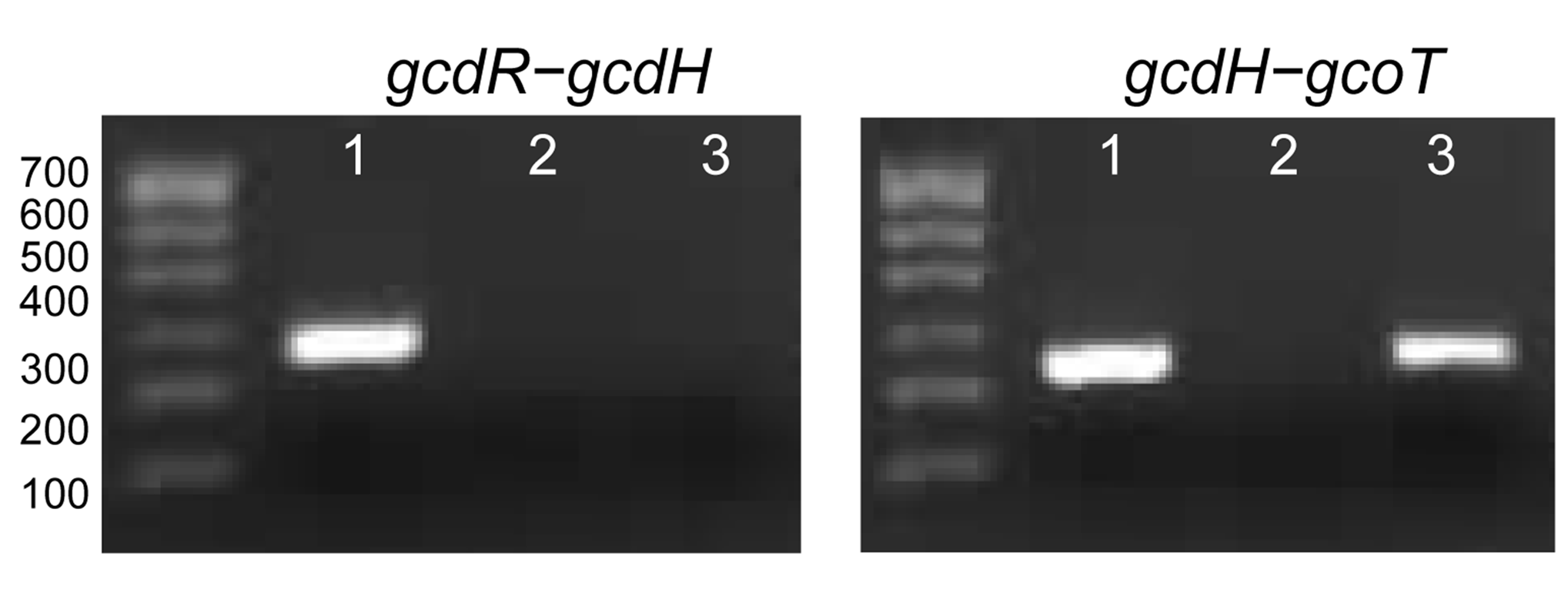

Supplement: FIG S4 [file mBio.01570-19-sf004.tif]

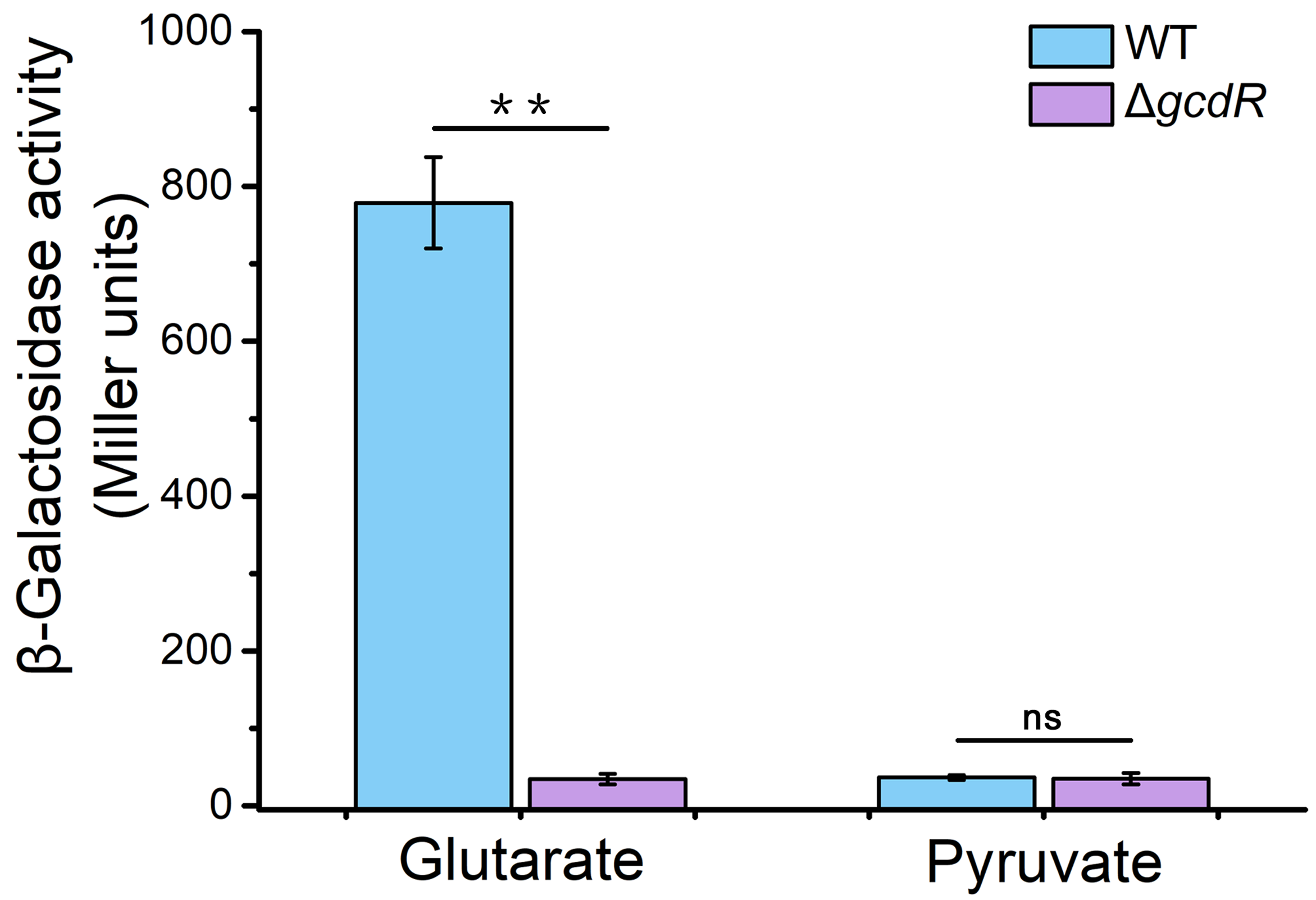

Supplement: FIG S5 [file mBio.01570-19-sf005.tif]

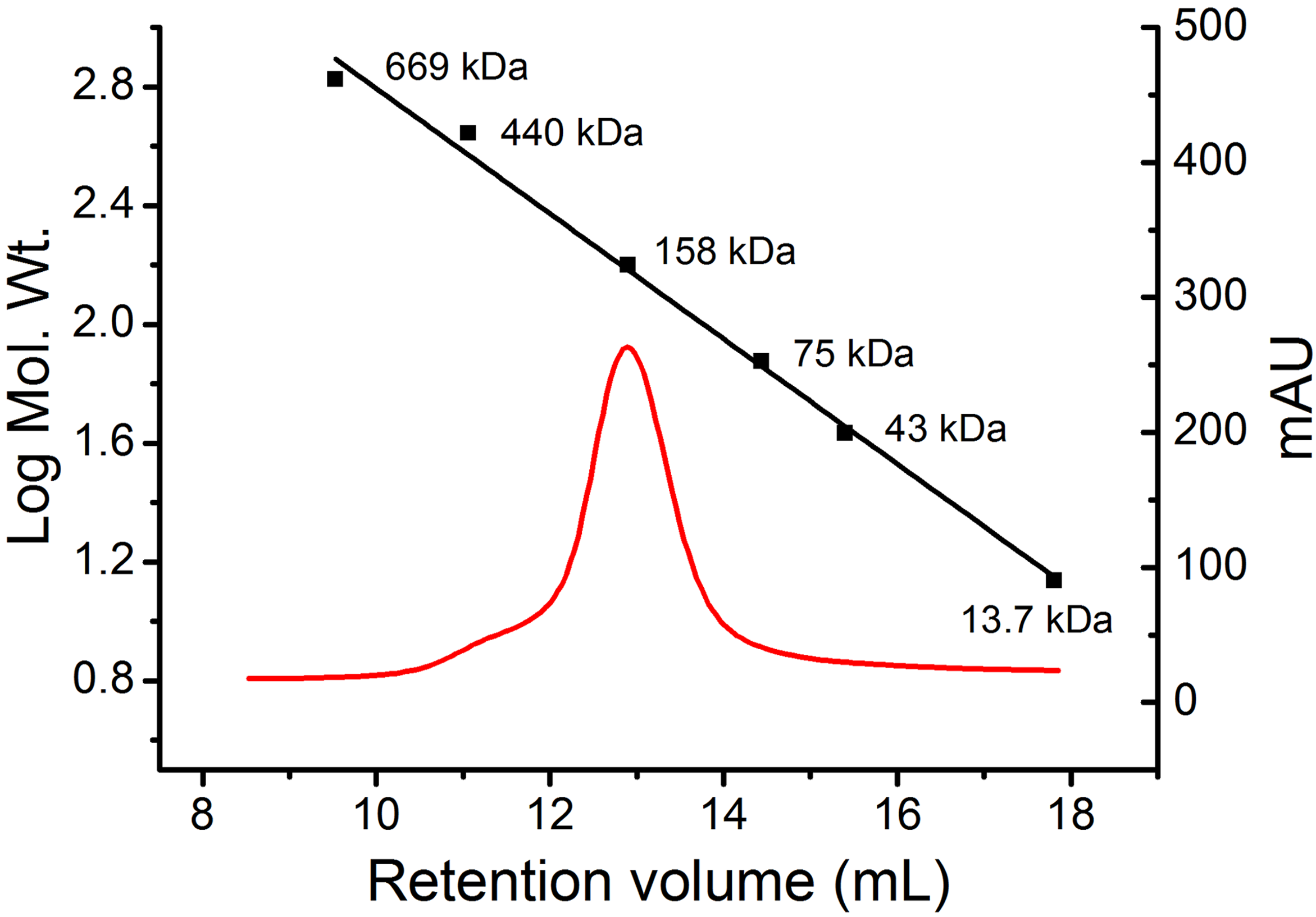

Supplement: FIG S6 [file mBio.01570-19-sf006.tif]

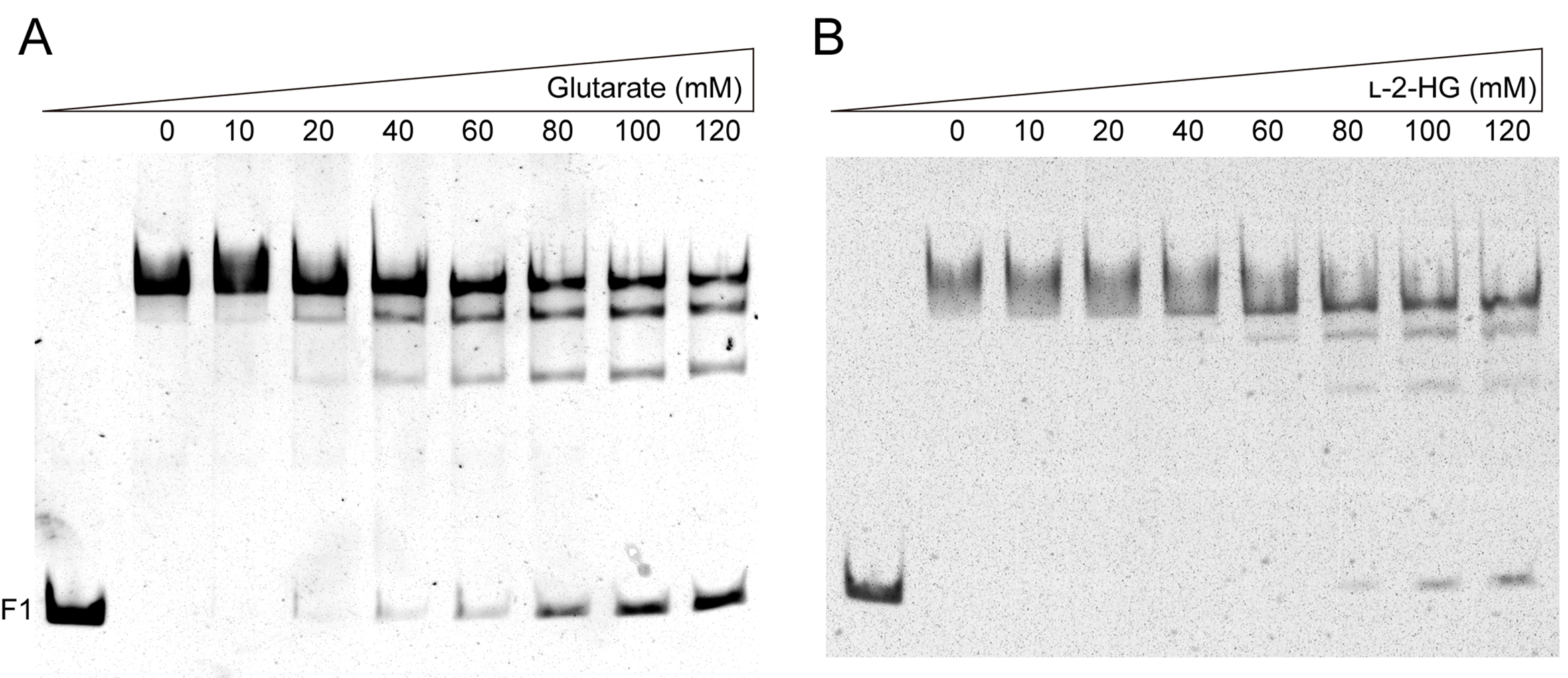

Supplement: FIG S7 [file mBio.01570-19-sf007.tif]

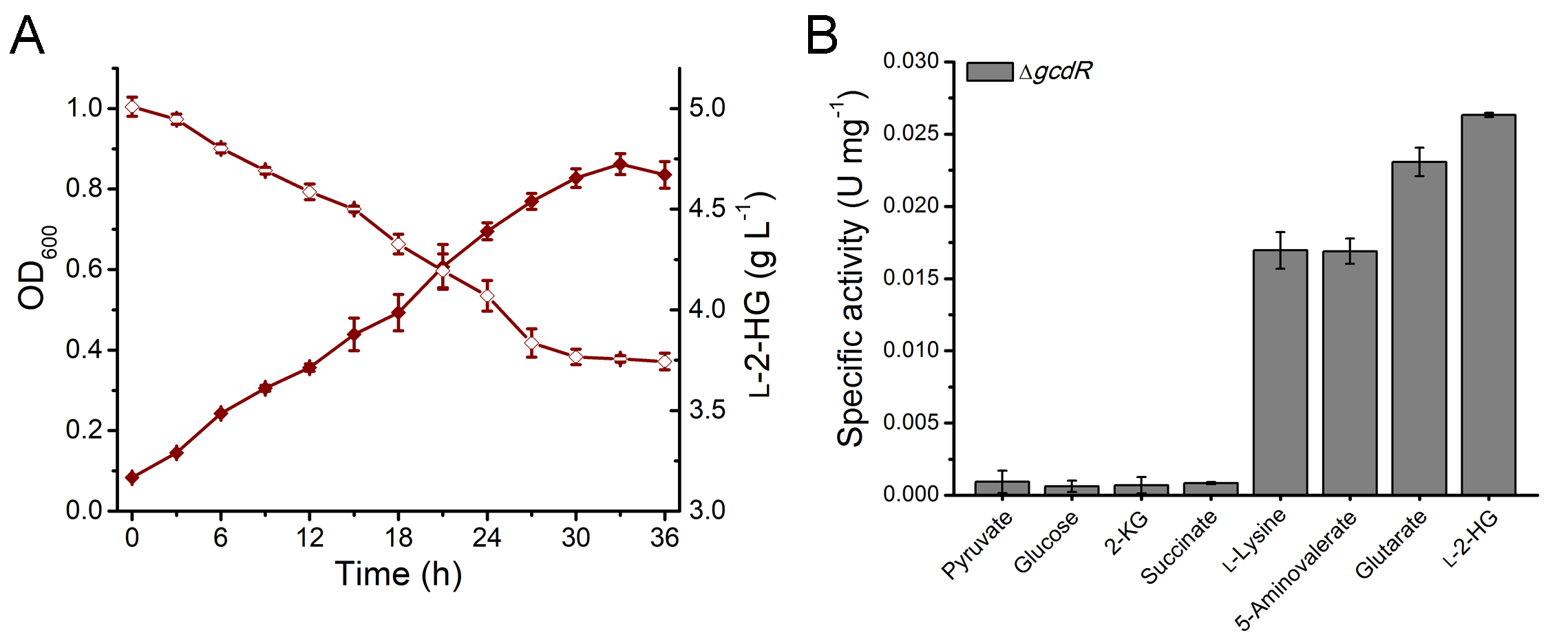

Supplement: FIG S8 [file mBio.01570-19-sf008.tif]
