# Supplementary material for: Regulation of Glutarate Catabolism by GntR Family Regulator CsiR and LysR Family Regulator GcdR in Pseudomonas putida KT2440
Source: mBio. 2019 Jul 30;10(4):e01570-19. doi: 10.1128/mBio.01570-19 (PMC6667623; doi:10.1128/mBio.01570-19)
Supplement: TABLE S1 [file mBio.01570-19-st001.doc]

**Table S1. Strains and plasmids used in this study**

| Strain or plasmid | Relevant characteristics^a^ |
| --- | --- |
| **Strain** |  |
| *P. putida* KT2440 | Wild-type |
| *P. putida* KT2440 (Δ*csiD*) | *P. putida* KT2440 mutant obtained by deletion of the *csiD* gene |
| *P. putida* KT2440 (Δ*csiR*) | *P. putida* KT2440 mutant obtained by deletion of the *csiR* gene |
| *P. putida* KT2440 (Δ*gcdH*) | *P. putida* KT2440 mutant obtained by deletion of the *gcdH* gene |
| *P. putida* KT2440 (Δ*gcdR*) | *P. putida* KT2440 mutant obtained by deletion of the *gcdR* gene |
| *P. putida* KT2440 (Δ*csiD*Δ*gcdR*) | *P. putida* KT2440 mutant obtained by deletion of the *csiD* gene and *gcdR* gene |
| *P. putida* KT2440 (Δ*gcdH*Δ*gcdR*) | *P. putida* KT2440 mutant obtained by deletion of the *gcdH* gene and *gcdR* gene |
| *P. putida* KT2440 (Δ*davT*Δ*alr*) | *P. putida* KT2440 mutant obtained by deletion of the *davT* gene and *alr* gene |
| *P. putida* KT2440 (Δ*lhgO*) | *P. putida* KT2440 mutant obtained by deletion of the *lhgO* gene |
| WT-pME6522-*P_csiD_* | *P. putida* KT2440 harboring the plasmid pME6522-*P_csiD_* |
| WT-pME6522-*P_gcdH_* | *P. putida* KT2440 harboring the plasmid pME6522-*P_gcdH_* |
| Δ*davT*Δ*alr*-pME6522-*P_csiD_* | *P. putida* KT2440 (Δ*davT*Δ*alr*) harboring the plasmid pME6522-*P_csiD_* |
| Δ*davT*Δ*alr*-pME6522-*P_gcdH_* | *P. putida* KT2440 (Δ*davT*Δ*alr*) harboring the plasmid pME6522-*P_gcdH_* |
| *E. coli* DH5α | F^–^ φ80*lacZ*∆M15 ∆(*lacZYA-argF*)U169 *deoR recA*1 *endA*1 *hsdR*17(r_K_^–^, m_K_^+^) *phoA* *supE*44 λ^–^ *thi-*1 *gyrA*96 *relA*1, used for gene clone |
| *E. coli* BL21(DE3) | F^–^ *ompT hsdSB*(*rB- mB-*) *gal*(λ *c I* 857 *ind1 Sam*7 *nin*5 *lac*UV5-T7*gene*1) *dcm* (DE3) |
| BL21-CsiR | *E. coli* BL21(DE3) harboring the expression plasmid pETDuet-*csiR* |
| BL21-GcdR | *E. coli* BL21(DE3) harboring the expression plasmid pET28a-*gcdR* |
| **Plasmid** |  |
| pETDuet-1 | Vector for protein expression; Ap^r^ |
| pETDuet-*csiR* | pETDuet-1 contained *csiR* gene of *P. putida* KT2440 |
| pET28a | Vector for protein expression; Km^r^ |
| pET28a-*gcdR* | pET28a contained *gcdR* gene of *P. putida* KT2440 |
| pMD18-T | TA Cloning vector, Ap^r^ |
| pK18*mobsacB* | Suicide plasmid for gene knockout; Km^r^ |
| pK18*mobsacB*-Δ*lhgO* | Partial lengths of *lhgO* were inserted into pK18*mobsacB* |
| pK18*mobsacB*-Δ*csiR* | Partial lengths of *csiR* were inserted into pK18*mobsacB* |
| pK18*mobsacB*-Δ*gcdH* | Partial lengths of *gcdH* were inserted into pK18*mobsacB* |
| pK18*mobsacB*-Δ*gcdR* | Partial lengths of *gcdR* were inserted into pK18*mobsacB* |
| pK18*mobsacB*-Δ*davT* | Partial lengths of *davT* were inserted into pK18*mobsacB* |
| pK18*mobsacB*-Δ*alr* | Partial lengths of *alr* were inserted into pK18*mobsacB* |
| pME6522 | pVS1-p15A *E. coli*-*Pseudomonas* shuttle vector for transcriptional *lacZ* fusion and promoter probing, Tc^r^ |
| pME6522-*P_csiD_* | 134-bp fragment, upstream of TSS of *csiD* operon, directionally cloned into pME6522 |
| pME6522-*P_gcdH_* | 124-bp fragment, upstream of TSS of *gcdH* operon, directionally cloned into pME6522 |
| pEASY-Blunt cloning vector | Vector for gene cloning; Ap^r^ |
| pEASY-Blunt-F1 | pEASY-Blunt cloning vector with 195-bp fragment of *csiR*-*csiD* intergenic region |
| pEASY-Blunt-F2 | pEASY-Blunt cloning vector with 260-bp fragment of *gcdR*-*gcdH* intergenic region and 50-bp extension upstream and downstream |

^a^Ap^r^, ampicillin resistant; Km^r^, kanamycin resistant; Tc^r^, tetracycline resistant.
