# Supplementary material for: Regulation of Glutarate Catabolism by GntR Family Regulator CsiR and LysR Family Regulator GcdR in Pseudomonas putida KT2440
Source: mBio. 2019 Jul 30;10(4):e01570-19. doi: 10.1128/mBio.01570-19 (PMC6667623; doi:10.1128/mBio.01570-19)
Supplement: TABLE S2 [file mBio.01570-19-st002.doc]

**Table S2. Primers used in this study**

| Primer | Sequence(5’-3’)^a^ | | Use |
| --- | --- | --- | --- |
| **Overexpression** | | | |
| *csiR*-F | | ATAGGATCCGTTGGAAGCGCTCGCCCCCCG (BamHI) | Amplification of *csiR* in *P. putida* KT2440 (forward) |
| *csiR*-R | | ATAAAGCTTCTAGCGGGCACCGATGCCTT (HindIII) | Amplification of *csiR* in *P. putida* KT2440 (reverse) |
| *gcdR*-F | | AATTCCATGGGCATGCGCCGTAAGATCC (NcoI) | Amplification of *gcdR* in *P. putida* KT2440 (forward) |
| *gcdR*-R | | AATTAAGCTTATTTGCCGCGTTTGTC (HindIII) | Amplification of *gcdR* in *P. putida* KT2440 (reverse) |
| **Gene knockout** | | | |
| *lhgO*-uf | | ATTGAATTCGGTGTACGATTTCATCATCATT (EcoRI) | Amplification of upstream homologous arm of *lhgO* (forward) |
| *lhgO*-ur | | CAGGTCGCTGGGGCTACCGACGATGTTGGGCT | Amplification of upstream homologous arm of *lhgO* (reverse) |
| *lhgO*-df | | CAACATCGTCGGTAGCCCCAGCGACCTGTTCC | Amplification of downstream homologous arm of *lhgO* (forward) |
| *lhgO*-dr | | AATAAGCTTTCAGCTGGCAGCCCTCTGGTTG (HindIII) | Amplification of downstream homologous arm of *lhgO* (reverse) |
| *csiR*-uf | | ATAGGATCCGATCTGATTGTGCCAAAGAA (BamHI) | Amplification of upstream homologous arm of *csiR* (forward) |
| *csiR*-ur | | TGTGGGCCCGCTCTCCAAGCACCTG | Amplification of upstream homologous arm of *csiR* (reverse) |
| *csiR*-df | | CAGGTGCTTGGAGAGCGGGCCCACA | Amplification of downstream homologous arm of *csiR* (forward) |
| *csiR*-dr | | ATAGAATTCCATGACCTGGATCAGTGCG (EcoRI) | Amplification of downstream homologous arm of *csiR* (reverse) |
| *gcdR*-uf | | ATAGAATTCATGCGCCGTAAGATCCCCAG (EcoRI) | Amplification of upstream homologous arm of *gcdR* (forward) |
| *gcdR*-ur | | ATTCATGCCCACATACCGCCAGCTC | Amplification of upstream homologous arm of *gcdR* (reverse) |
| *gcdR*-df | | GAGCTGGCGGTATGTGGGCATGAAT | Amplification of downstream homologous arm of *gcdR* (forward) |
| *gcdR*-dr | | ATAGGATCCTCAATTTGCCGCGTTTGTCC (BamHI) | Amplification of downstream homologous arm of *gcdR* (reverse) |
| *davT*-uf | | CGCGGATCCATGAGCAAAACCAACGA (BamHI) | Amplification of upstream homologous arm of *davT* (forward) |
| *davT*-ur | | ATTCGGCCTTGCACGGGCGATCTT | Amplification of upstream homologous arm of *davT* (reverse) |
| *davT*-df | | AAGATCGCCCGTGCAAGGCCGAAT | Amplification of downstream homologous arm of *davT* (forward) |
| *davT*-dr | | GCGAAGCTTTCAGGCGATTTCAGCGA (Hind III) | Amplification of downstream homologous arm of *davT* (reverse) |
| *alr*-uf | | GCGAAGCTTTCAGTCGACGAGTATCT (HindIII) | Amplification of upstream homologous arm of *alr* (forward) |
| *alr*-ur | | GACATGGAAGAGCCGAGTACAAACG | Amplification of upstream homologous arm of *alr* (reverse) |
| *alr*-df | | CGTTTGTACTCGGCTCTTCCATGTC | Amplification of downstream homologous arm of *alr* (forward) |
| *alr*-dr | | ATAGGATCCATGCCCTTTCGCCGTAC (BamHI) | Amplification of downstream homologous arm of *alr* (reverse) |
| **RACE-PCR** | | | |
| *csiD*-GSP1 | | ATCAGGTGAGCGCAGGCGGTGGTG | Oligonucleotide for reverse transcription of *csiD* |
| *csiD*-GSP2 | | TTCGGCCTGGCTCACGTCATCC | Oligonucleotide for first round PCR of the 5′ RACE of *csiD* |
| *csiD*-GSP3 | | TGGCGCGGTCGAGGATGGTGTTG | Oligonucleotide for nested PCR of the 5′ RACE of *csiD* |
| *gcdH*-GSP1 | | ATCCACTCGCCACTGGCAAGCT | Oligonucleotide for reverse transcription of *gcdH* |
| *gcdH*-GSP2 | | TCGGTGCCAAATTCGTTGATCGG | Oligonucleotide for first round PCR of the 5′ RACE of *gcdH* |
| *gcdH*-GSP3 | | CGAGCGGTAACCCGAGTCGATGCGT | Oligonucleotide for nested PCR of the 5′ RACE of *gcdH* |
| ***lacZ* fusion** | |  |  |
| *P_csiD_*-F | | AATTGAATTCGATCCTGTTCCTCAAAACGT (EcoRI) | Amplification of a 134-bp fragment *P_csiD_* (forward) |
| *P_csiD_-*R | | AATTCTGCAGCGAGGTAAAAATATCGCCACT (PstI) | Amplification of a 134-bp fragment *P_csiD_* (reverse) |
| *P_gcdH_*-F | | AATTGAATTCGTACGTCAACCTCACTTGTAA (EcoRI) | Amplification of a 124-bp fragment *P_gcdH_* (forward) |
| *P_gcdH_*-R | | AATTCTGCAGCTTTGAGCCTAGGCCTGATCG (PstI) | Amplification of a 124-bp fragment *P_gcdH_* (reverse) |
| **EMSAs** | |  |  |
| F1-F | | GATCCTGTTCCTCAAAACG | Amplification of fragment F1 (forward) |
| F1-R | | GGCAGTGTCCTGGCTAAAA | Amplification of fragment F1 (reverse) |
| F2-F | | TGATCCAGCAGCAACGGG | Amplification of fragment F2 (forward) |
| F2-R | | GCCGCCTCGAAGCAAATCA | Amplification of fragment F2 (reverse) |
| C-F | | CGTTCTTCCGTCACCCTCTGG | Amplification of fragment C (forward) |
| C-R | | GGCTGCACGAACTGGTCAATG | Amplification of fragment C (reverse) |

^a^Restriction sites are underlined, and the restriction enzymes are indicated in parentheses.
